# Dietary Oligofructose Alone or in Combination with 2′-Fucosyllactose Differentially Improves Recognition Memory and Hippocampal mRNA Expression

**DOI:** 10.3390/nu12072131

**Published:** 2020-07-17

**Authors:** Stephen A. Fleming, Austin T. Mudd, Jonas Hauser, Jian Yan, Sylviane Metairon, Pascal Steiner, Sharon M. Donovan, Ryan N. Dilger

**Affiliations:** 1 Piglet Nutrition and Cognition Laboratory, Department of Animal Sciences, University of Illinois, Urbana, IL 61801, USA; stephen@traversescience.com (S.A.F.); mudd.austin@gmail.com (A.T.M.); 2 Neuroscience Program, University of Illinois, Urbana, IL 61801, USA; 3 Nestlé Research, 1000 Lausanne, Switzerland; Jonas.Hauser@rdls.nestle.com (J.H.); Sylviane.Metairon@rd.nestle.com (S.M.); Pascal.Steiner@rdls.nestle.com (P.S.); 4 Nestlé Product Technology Center Nutrition, 3510 Konolfingen, Switzerland; jy435@cornell.edu; 5 Department of Food Science and Human Nutrition, University of Illinois, Urbana, IL 61801, USA; sdonovan@illinois.edu; 6 Division of Nutritional Sciences, University of Illinois, Urbana, IL 61801, USA

**Keywords:** prebiotics, milk, pig, brain, cognition, neuroimaging, human milk oligosaccharides, infant, nutrition

## Abstract

Mounting evidence suggests that dietary oligosaccharides promote brain development. This study assessed the capacity of oligofructose (OF) alone or in combination with 2′-fucosyllactose (2′-FL) to alter recognition memory, structural brain development, and hippocampal gene expression. Beginning on postnatal day (PND) 2, male pigs received one of three milk replacers formulated to contain OF, OF + 2′-FL, or no oligosaccharides (CON). Pigs were tested on the novel object recognition task using delays of 1 or 48 h at PND 22. At PND 32–33, magnetic resonance imaging (MRI) procedures were used to assess structural brain development and hippocampal tissue was collected for analysis of mRNA expression. Pigs that consumed the OF diet demonstrated increased recognition memory after a 1 h delay, whereas those consuming diets containing OF + 2′-FL displayed increased recognition memory after a 48 h delay. Pigs fed OF or OF + 2′-FL exhibited a larger relative volume of the olfactory bulbs compared with CON pigs. Provision of OF or OF + 2′-FL altered gene expression related to dopaminergic, GABAergic, cholinergic, cell adhesion, and chromatin remodeling processes. Collectively, these data indicate that dietary OF and OF + 2′-FL differentially improve cognitive performance and affect olfactory bulb structural development and hippocampal gene expression.

## 1. Introduction

While their ability to stimulate gut bacteria and prebiotic activity has been known for some time, it is becoming increasingly evident that oligosaccharides (OS) act through unknown mechanisms to stimulate brain development. Oligofructose (OF), also known as fructooligosaccharide (FOS), is an OS of vegetable origin, commonly found in foods such as asparagus, artichoke, onion, and wheat [1]. Oligofructose is non-digestible and readily fermentable by *Bifidobacterium* spp. and *Bacteroides* spp., but not by potentially pathogenic bacteria such as *Escherichia coli* and *Clostridium perfringens* [2]. Infants consuming formula containing OF display increased fecal bifidobacteria and bacteroides, decreased fecal *Escherichia coli* and enterococci, and increased stool frequency [3,4]. Consumption of OF has also been shown to alter the expression of brain-derived neurotrophic factor (BDNF) and *N*-methyl-D-aspartate (NMDA) receptor subunits in the rodent brain [5] and improve cognitive function in a rodent model of Alzheimer’s disease [6,7].

Human milk contains a heterogenous group of OS that have demonstrated benefits for immune and intestinal function and are hypothesized to promote brain development [8,9]. The concentration and diversity of these human milk oligosaccharides (HMO) is unmatched by other mammals [10], and these OS are specific to milk as opposed to non-milk OS such as OF. This has significant implications for infants consuming bovine milk-based infant formulas, as bovine milk contains up to a hundred times fewer and less diverse OS [9]. Infants consuming formula containing HMO such as 2′-fucosyllactose (2′-FL) and Lacto-*N*-neotetraose report fewer incidences of respiratory illness [11]. Evidence from rodent studies suggests that HMO such as sialyllactose and 2′-FL may improve response to stress [12] and learning and memory [13,14], respectively. Both sialyllactose and 2′-FL contain monosaccharides (i.e., sialic acid and fucose, respectively) that are known glycoconjugates in the brain [15,16]. Whether sialyllactose and 2′-FL impact the brain in large part due to their sialic acid or fucose content is unclear. Sialic acid, alone or as part of a ganglioside, is known to promote cognition [17,18,19]. Similarly, fucose has been shown to accrete in glycoproteins after a passive avoidance task in chicks [16], and impairing fucosylation in the rat hippocampus impairs retention during discrimination tasks [20]. Yet, Vazquez et al. demonstrated that intact 2′-FL, and not fucose, promotes hippocampal long-term potentiation [14].

Alluding to the probability that intact OS and not their active monosaccharide components are required for promoting cognition is the evidence that OS such as OF, galactooligosaccharide (GOS), and chitosan oligosaccharide have been shown to benefit cognition in various animal models and species [7,21,22,23]. This is significant as some formulas contain OF or GOS [24], yet these OS are not found in human milk. Whether HMO provide a cognitive benefit greater than that of non-human milk OS is an important question as infants relying on formula as their sole source of nutrition are typically not provided the level and diversity of HMO that are present in human milk.

We chose to use the neonatal pig as an animal model due to fact that similarities with the human regarding gastrointestinal physiology [25], brain development [26], and strengths and limitations of comparisons to the human microbiome are well described [27,28,29]. As the majority of studies assessing the efficacy of OS to promote brain development have been conducted in rodent models, this study evaluated whether such effects can be replicated in an animal model closer to humans. As previously mentioned, it is unclear whether HMO provide a cognitive benefit in addition to formula already containing non-HMO OS. Thus, the objective of this study was to assess the impact of dietary OF alone or combined with 2′-FL on recognition memory, hippocampal gene expression, and structural development of the brain using the pig as an animal model.

## 2. Materials and Methods

### 2.1. Animals and Housing

All animal care and experimental procedures were in accordance with the National Research Council Guide for Care and Use of Laboratory Animals and approved by the University of Illinois at Urbana-Champaign Institutional Animal Care and Use Committee (IACUC 15034). In general, rearing and housing methods were conducted similarly to previous studies from our lab [21], and are described as follows. Thirty-six intact male pigs (1050 Cambro genetics) were naturally farrowed and allowed colostrum consumption for up to 48 h before transport to the Piglet Nutrition and Cognition Laboratory at the University of Illinois at Urbana-Champaign. Pigs were artificially reared from postnatal day (PND) 2 until PND 33. This study was conducted using six independent cohorts (*n* = 2 pigs per dietary treatment in each cohort), with litter and initial bodyweight counterbalanced between dietary groups and within each cohort. All pigs were housed in master caging units that contained six individual stainless-steel cages (L × W × H of 87.6 × 88.9 × 50.8 cm), with clear, polycarbonate facades on three sides of the cage and vinyl-coated, expanded-metal flooring (Tenderfoot ^®^, Minneapolis, MN, USA). The master unit was designed such that there were three separate levels each with two individual pig cages on each level. Thus, pigs on each level shared a common wall containing holes to permit pigs to see, smell, hear, and minimally touch one another. A towel and toy were included in each cage to provide enrichment, all pigs were removed from cages and allowed to socialize for approximately 30 min each day, and all pigs were allowed *ad libitum* access to water at all times.

**Figure 1:**
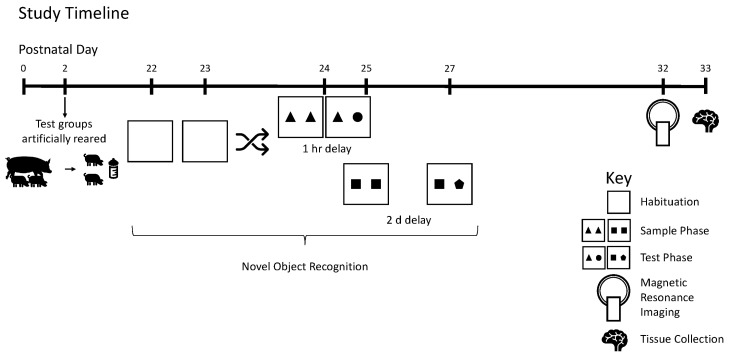
Study timeline. Pigs were reared artificially from postnatal days 2–33. On postnatal day 22, pigs were tested on the novel object recognition test twice using delays of 1 h or 2 days, with task order randomized and counterbalanced between groups. On PND 32–33, pigs were subjected to magnetic resonance imaging; and on PND 33, brain tissue was collected for quantification of hippocampal gene expression. Pigs were weighed daily to track growth.

All pigs were reared in the same room with ambient temperature maintained between 27 and 29 °C and a 12 h light/dark cycle maintained from 600 to 1800 h. Prior to placement in the artificial rearing system, pigs were administered 5.0 mL of *Clostridium perfingens* antitoxin C + D per the manufacturer’s recommendations (Colorado Serum Company, Denver, CO, USA) to prevent enterotoxemia [30]. At study conclusion (PND 33), pigs were anesthetized using a telazol: ketamine: xylazine solution (50.0 mg tiletamine plus 50.0 mg of zolazepam reconstituted with 2.50 mL ketamine [100 g/L] and 2.50 mL xylazine [100 g/L]; Fort Dodge Animal Health) by intramuscular injection at 0.03 mL/kg bodyweight. After anesthetic induction, pigs were euthanized via intracardiac administration of sodium pentobarbital (86.0 mg/kg of body weight; Euthasol, Virbac Animal Health, Fort Worth, TX, USA). Pigs were observed twice daily at approximately 800 and 1600 h and given health scores to track any weight loss, vomiting, diarrhea, or lethargic behavior. Figure 1 demonstrates the study design.

### 2.2. Dietary Treatments

All researchers involved with conducting the study and acquiring and analyzing study results remained blind to dietary treatment identity until final data analyses were complete. Pigs (*n* = 12 per diet) were provided milk replacers reconstituted at 200 g of dry powder per 800 g of water. Reconstituted diets were formulated to contain approximately 0 g/L OF + 0 g/L 2′-FL (control [CON], ProNurse^®^ Specialty Milk Replacer, Purina Animal Nutrition, Gray Summit, MO, USA), 5 g/L OF + 0 g/L 2′-FL (OF), or 5 g/L OF + 1 g/L 2′-FL (OF + 2′-FL). The concentration of oligosaccharides was chosen to remain consistent with previous clinical studies investigating stool characteristics infants of fed formula containing 3–5 g/L oligofructose [31,32] or impact on growth of infants fed formulas containing 1 g/L 2′-fucosyllactose [11,33]. As the aim of the research was to assess the addition of 2′-FL to a diet already containing an oligosaccharide source, a group fed 2′-FL alone was not included. The base diet was diluted to allow the addition of oligosaccharides and all diets were formulated to contain supplemental lactose to balance the amount of total carbohydrate. Thus, diets contained equivalent fat and protein content with as minimal as possible adjustments to the lactose content. Nutrient composition of the base diet and both formulated and analyzed concentrations of oligosaccharides and lactose in the diet are shown in Table 1 and Table 2.

Pigs received small volumes (approximately 500 mL) of experimental diets on the day of arrival to the rearing facility to allow for adjustment to the milk replacer prior to the standard feeding regimen. Pigs were fed at a rate of 285 and 325 mL of reconstituted diet per kg bodyweight from PND 3–6 and PND 7–33, respectively. Individual pig bodyweight was recorded daily to determine the volume of milk to be dispensed to individual animals throughout the day. Meals were administered 10 times a day, approximately every 100 min, between 400 and 1000 h using an automated feeding system. Feed refusals were not quantified.

### 2.3. Behavior

Pigs were tested on the novel object recognition (NOR) task using two different delays to assess intermediate and long-term recognition memory. This task has previously been validated for use in pigs by independent labs [34,35,36,37] and the methods used for execution and analysis of this test by our lab have been previously described [21,38,39]. Testing consisted of a habituation phase, a sample phase, and a test phase. During the habituation phase, each pig was placed in an empty testing arena for 10 min each day for two days leading up to the sample phase. In the sample phase, the pig was placed in the arena containing two identical objects and given 5 min for exploration. After a delay of 1 or 48 h the pig was returned to the arena for the test phase of the NOR task. During the test phase, the pig was placed in the arena containing one object from the sample phase and a novel object and allowed to explore for 5 min. Between trials, objects were removed, immersed in hot water with detergent, and rubbed with a towel to mitigate odor and the arena was sprayed with water to remove urine and feces. Objects chosen had a range of characteristics (i.e., color, texture, shape, and size). However, the novel and sample objects only differed in shape and size. Only objects previously shown to elicit a null preference were used for testing [35]. Task order was counterbalanced between replicates. Habituation trials began at PND 22 and testing on the sample phase began on PND 24. The recognition index, or the proportion of time spent with the novel object compared to total exploration of both objects, was used to measure recognition memory. A recognition index significantly above 0.50 demonstrates a novelty preference and thus recognition memory. Videos from all experiments were analyzed using a commercially available software package (Ethovision XT 11^®^, Noldus Information Technology, Wageningen, The Netherlands). Time spent investigating objects was recorded manually by mapping start and stop conditions to specific keys on a computer keyboard. Experimenters were blind to all treatment conditions during analysis. Investigations were classified as nose-directed behavior such as rooting, mouthing, or sniffing of the objects. Rubbing up against, standing over, standing near, looking at, or sniffing the floor/air near the objects were not counted as investigations.

### 2.4. Magnetic Resonance Imaging (MRI)

All pigs underwent MRI procedures at PND 32 or 33 at the Beckman Institute for Advanced Science and Technology Biomedical Imaging Center using Siemens MAGNETOM Trio 3T (Siemens, Munich, Germany) equipment with a Siemens 32-channel head coil. Methods used were adapted from previous studies using MRI in pig [40,41,42]. Each pig underwent imaging protocols only once, and scans for each cohort of pigs were completed all on the same day. The pig neuroimaging protocol included three magnetization prepared rapid gradient-echo (MPRAGE) sequences and diffusion tensor imaging (DTI) to assess brain macrostructure and microstructure, respectively, as well as magnetic resonance spectroscopy (MRS) to obtain brain metabolite concentrations. In preparation for MRI procedures, anesthesia was induced using an intramuscular injection of telazol (50.0 mg of tiletamine plus 50.0 mg of zolazepam reconstituted with 5.0 DI water; Zoetis, Florham Park, NJ) administered at 0.07 mL/kg bodyweight, and maintained with inhalation of isoflurane (98% O_2_, 2% isoflurane). Pigs were immobilized during all MRI procedures. Visual observation of each pig’s well-being, as well as observations of heart rate, PO_2_ and percent of isoflurane were recorded every 5 min during the procedure and every 10 min post-procedure until animals recovered. Total scan time for each pig was approximately 60 min. Imaging techniques are briefly described below.

#### 2.4.1. Structural MRI

A T_1_-weighted magnetization-prepared rapid gradient echo (MPRAGE) sequence was used to obtain anatomic images of the pig brain with a 0.7 mm isotropic voxel size. Three repetitions were acquired and averaged using SPM8 in Matlab 8.3, and brains were manually extracted using FMRIB Software Library (FSL) (FMRIB Centre, Oxford, UK). Manual extraction was initially performed by a single trained observer and reviewed by a second trained observer blind to experimental treatment. The following sequence specific parameters were used to acquire T_1_-weighted MPRAGE data: repetition time (TR) = 1900 ms; echo time (TE) = 2.49 ms; 224 slices; field of view (FOV) = 180 mm; flip angle = 9°. Methods for MPRAGE averaging and manual brain extraction were previously described [42]. All data generated used a publicly-available population-averaged pig brain atlas (http://pigmri.illinois.edu) [43]. For volumetric assessments, individual brains were segmented into 22 different regions of interest (ROI) using the pig brain atlas. Total brain and individual region volume analysis was performed with SPM8 in which an inverse warp file for each ROI was generated from the DARTEL-generated warp files for each region. As described previously [42], the SPM ‘Segment’ tool, along with pig-specific tissue prior probabilities, was used to obtain gray matter, white matter, and CSF tissue segmentations for each pig, and DARTEL was used to align the native space segmentations. The fslstats toolbox was used to determine the voxel volume of the subject-space segmentation for each of the three tissue types. Using fslmaths, the mean overall partial volume map was obtained for each subject-space segmentation. Overall absolute volume for gray matter, white matter, and CSF was determined by multiplying the voxel volume measure by the mean intensity of the partial volume segmentation. In order to account for absolute whole-brain volume, all regions of interest were also expressed as a percent of total brain volume (%TBV).

#### 2.4.2. Diffusion Tensor Imaging

Diffusion tensor imaging was used to assess white matter maturation and axonal tract integrity using a *b*-value = 1000 s/mm^2^ across 30 directions and a 2 mm isotropic voxel. Diffusion-weighted echoplanar images (EPIs) were assessed in FSL 5.0 for fractional anisotropy (FA), mean diffusivity (MD), axial diffusivity (AD), and radial diffusivity (RD) using methods previously described [42]. The pig brain atlas was used for assessment of the following regions of interest: caudate, corpus callosum, cerebellum, both hippocampi, internal capsule, left and right cortex, thalamus, DTI-generated white matter, and atlas-generated white matter using a customized pig analysis pipeline and the FSL software package. The diffusion toolbox in FSL was used to generate values of AD, RD, MD, and FA. In the corresponding results, atlas-generated white matter indicates the use of white matter prior to using probability maps from the pig brain atlas that were used as a region of interest mask. Likewise, DTI-generated white matter indicates a threshold of 0.2 was applied to FA values, thus restricting analysis to white matter tracts. Masks for each ROI from the atlas were non-linearly transformed into the MPRAGE space of each individual pig and a linear transform was then applied to transfer each ROI into DTI space. A threshold of 0.5 was applied to each ROI, and the data were dilated twice. For each individual ROI, an FA threshold of 0.15 was applied to ensure that we included only white matter in that region of interest despite the mask expansion.

#### 2.4.3. Magnetic Resonance Spectroscopy

Magnetic resonance spectroscopy was used to non-invasively quantify metabolites in the whole brain. The MRS spin-echo chemical shift sequence was used with a voxel size of 20 mm × 23 mm × 13 mm and centered over the left and right dorsal hippocampi. The following sequence parameters were used in acquisition of spectroscopy data for the water-suppressed scan: TR = 1800 ms; TE = 68 ms; signal averages = 256; vector size = 1024. The following sequence parameters were used in acquisition of spectroscopy data for the non-water-suppressed scan: TR = 20,000 ms; TE = 68 ms; signal averages = 1; vector size = 1024 point. Both water-suppressed and non-water-suppressed data were collected in institutional units, and all MRS data were analyzed with LC Model (version 6.3) using methods previously described [41]. Limits were placed on MRS data for inclusion in the statistical analysis. Cramer–Rao lower bounds (i.e., % standard deviation) were calculated using LC Model and only metabolites with a standard deviation less than 20% were considered to have reliable quantitative results of absolute levels.

### 2.5. Hippocampal Gene Expression

Approximately 20 mg of hippocampal tissue was introduced in a Lysing Matrix D tube (MP Biomedicals, Santa Ana, CA, USA), placed on ice, and 650 µL of lysis buffer (Agencourt RNAdvance Tissue Kit, Beckman Coulter, Indianapolis, Indiana, USA) was added. Tubes were agitated for 2 × 1 min at speed 6 on FastPrep^®^-24 (MP Biomedicals, Santa Ana, CA, USA), and 400 µL of lysate was then extracted using the Agencourt RNAdvance Tissue Kit (Beckman Coulter, Indianapolis, IN, USA) following the manufacturer’s recommendations. RNA were quantified using the Quant-iT™ RiboGreen™ RNA Assay Kit (Invitrogen, Carlsbad, CA, USA) on a Spectramax M2 (Molecular Devices, Sunnyvale, CA, USA). RNA quality assessment was completed using a Fragment Analyzer 96 with Standard Sensitivity RNA Analysis Kit (15 nt) (Advanced Analytical Technologies, Inc., Ankeny, IA, USA). Relative mRNA copy number on 93 genes was quantified using the NanoString nCounter™ system (NanoString Technologies Inc., Seattle, WA, USA) according to the manufacturer’s instructions using 100 ng of RNA as the starting amount. Using nSolver software (Version 4.0, NanoString Technologies Inc., Seattle, WA, USA), background subtraction using the median of all eight negative controls was followed by positive control normalization using the geometric mean of six positive controls and housekeeping normalization using the geometric mean of six housekeeping genes.

### 2.6. Statistical Analysis

Data analysis was conducted using the GLIMMIX procedure of SAS Enterprise Guide 7.1 (SAS Institute, Cary, NC, USA). All data were subjected to a one-way analysis of variance to assess the effect of dietary treatment. The cohort of pigs was included in the model as a random variable. For all variables, observations with a studentized residual greater than |3| were considered outliers and removed from that variable only. Distribution of data was assessed visually using diagnostic plots (e.g., QQ plots). However, formal tests for normality were not conducted, given that ANOVA is robust towards non-normality. For behavioral data, pigs that exhibited little exploration of either object (i.e., less than 2 s of exploration of the sample or novel objects) were considered non-compliant and their recognition index was not measured in the test phase (final sample size: 1 h delay; CON, *n* = 9; OF, *n* = 12; OF + 2′-FL, *n* = 10; 2-d delay; CON, *n* = 10; OF, *n* = 11, OF + 2′-FL, *n* = 10), but all other exploration measures were included for those subjects. Inclusion of non-compliant animals tends to misrepresent the true effect of the task, as only animals that explore an object should be tested for memory of a previously explored object. Variables from the sample phases from the 1 and 48 h delay paradigms were averaged to create sample phase exploration measures (e.g., total time visiting objects during the sample phase in the 1 and 48 h delay were average to create a single measure). To test for recognition memory, a one-sample *t-*test was conducted comparing the recognition index to a null mean of 0.5. Groups with a mean recognition index significantly above 0.5 were considered to demonstrate recognition memory.

For individual brain region volume assessment, volume was expressed in both absolute (i.e., mm^3^) and relative units (i.e., regional volume as a proportion of total brain volume, within subject). Gene expression data were standardized (mean of zero and standard deviation of one) and centered by the control group, thus all scores for the control group are zero. Statistical significance was defined at *p* < 0.05 (insignificant results provided in Supplemental Tables). Post-hoc comparisons for mean separation were conducted with a Tukey adjustment, and data are represented as least square means. Correlations between significant outcomes (MRI or gene expression) gene expression and the recognition index were conducted using the Pearson correlation coefficient for each diet and linear regression was used to assess the diet independent relationship between outcomes. Sample sizes for all variables assessed can be found in the Supplemental Tables.

## 3. Results

### 3.1. Growth and Behavior

**Figure 2:**
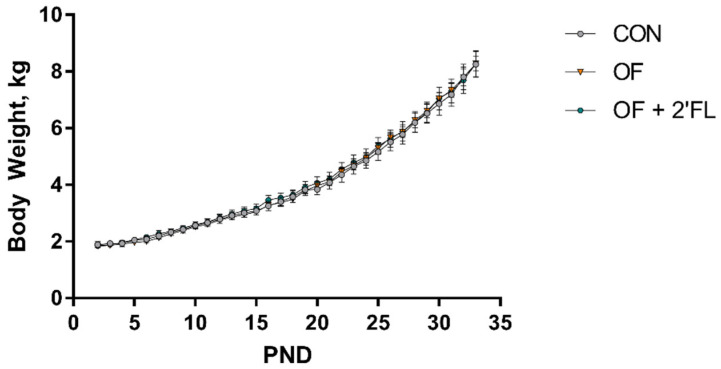
Body weight (BW) during the trial. No differences in average daily body weight gain were observed between groups (*p* = 0.99). Data are presented as the mean ± standard error.

**Figure 3:**
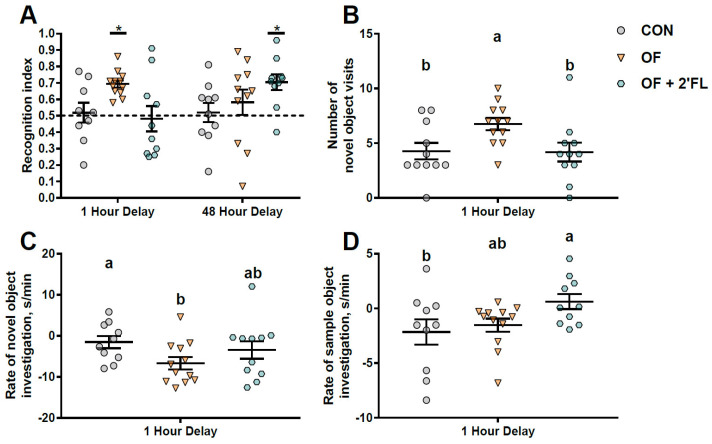
Recognition memory and exploratory behavior during the novel object recognition task. (**A**) The OF group was able to show recognition memory after a 1 h delay (one-sample *t-*test, *p* < 0.001). However, only the OF + 2′-FL group was able to show recognition memory after a 48 h delay (one-sample *t-*test, *p* = 0.001). Exploratory behavior was similar between groups after a 48 h delay. However, differences emerged after a 1 h delay. (**B**) The OF group visited the novel object more frequently than the CON group (*p* = 0.022), (**C**) whereas the control group maintained a high rate of exploration of the novel object throughout the trial (*p* = 0.045). That is, the OF group habituated to the novel object more quickly than the CON group. (**D**) On the contrary, exploration of the sample object by the OF + 2′-FL group increased as the trial went on compared to the CON group (*p* = 0.038). Lines depict the mean ± standard error, and groups without a common superscript differ (*p* < 0.05). Asterisks depict that a given group mean was significantly greater than 0.50, as measured by a one-sample *t-*test. (*p* < 0.05) Abbreviations: CON, control group; OF, pigs fed oligofructose; OF + 2′-FL, pigs fed oligofructose and 2’ fucosyllactose.

No difference in average daily body weight gain over the course of the study (*p* = 0.99, Figure 2) was found. Given that all animals were fed on a by-weight basis, daily body weight data suggest that pigs across treatments consumed similar amounts of milk replacer per day (milk intake not quantified). The control group failed to exhibit recognition memory after either 1 or 48 h delay. The OF group was able to show recognition memory after a 1 h delay (one-sample *t*-test, *p* < 0.001) but not after a 48 h delay (one-sample *t*-test, *p* = 0.155). On the other hand, the OF + 2′-FL group failed to show recognition memory after 1 h delay (one-sample t-test, *p* = 0.592), but was able to show recognition after a 48 h delay (one-sample *t-*test, *p* = 0.001, Figure 3A). Exploratory behaviors (e.g., distance moved, time spent exploring objects, frequency of object visits, and mean length of object visits) were similar between groups during both habituation trials, the sample trials, and the 48 h delay test trial (Supplemental Table S1). After a 1 h delay, the OF group demonstrated more frequent visits to the novel object compared with the CON group (*p* = 0.022), whereas the control group maintained a high rate of exploration of the novel object throughout the trial (*p* = 0.045). On the contrary, exploration of the sample object by the OF + 2′-FL group increased (pigs exhibited a positive rate of exploration [seconds exploring/minute]) as the trial went on compared to the CON group (*p* = 0.038, Figure 3).

### 3.2. MRI

**Figure 4:**
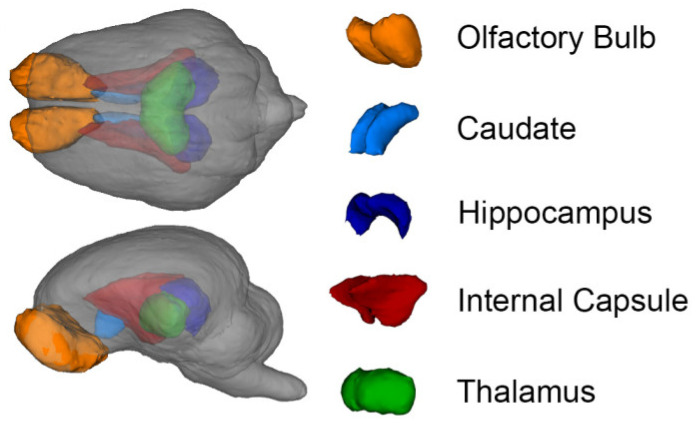
Representative 3D surface rendering of the brain from the pig brain atlas highlighting regions affected by diet.

**Figure 5:**
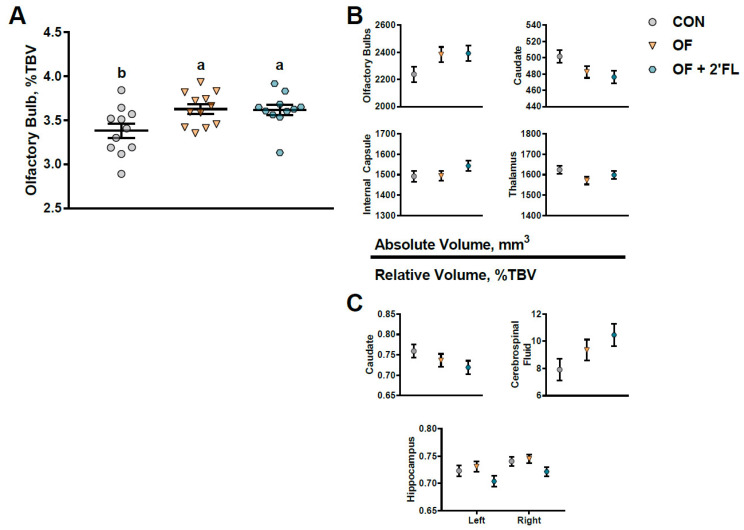
(**A**) Both the OF and OF + 2′-FL groups demonstrated larger relative volumes of the olfactory bulbs (*p* = 0.019) as compared to controls. (**B**) Trending effects of diet are shown for both absolute and (**C**) relative brain volumes (0.05 < *p* < 0.10). Lines depict the mean ± standard error, and groups without a common superscript differ (*p* < 0.05). Abbreviations: CON, control group; OF, pigs fed oligofructose; OF + 2′-FL, pigs fed oligofructose and 2′ fucosyllactose; %TBV, percent of total brain volume.

A 3D surface rendering of the brain regions affected by the diet is shown in Figure 4. Trending effects of diet were observed for the absolute volumes of the olfactory bulbs, caudate, internal capsule, and thalamus, whereas the relative volumes of the caudate, cerebrospinal fluid, and both hippocampi exhibited a trending effect of the diet (0.05 < *p* < 0.10, Figure 5, Supplemental Tables S2 and S3). The only significant effect observed was an increase in the relative volume of the olfactory bulbs in the OF and OF + 2′-FL groups compared with the CON group (*p* = 0.019, Figure 5). There was no effect of diet for any DTI measure (AD, MD, RD, or FA, all *p* > 0.165, Supplemental Tables S4 and S5). Of the MRS outcomes, only glutathione, myo-inositol, *N*-acetylaspartate, and γ-amino butyric acid met criteria for inclusion. However, none were altered by diet (all *p* > 0.303, Supplemental Table S6).

### 3.3. Hippocampal Gene Expression

**Figure 6:**
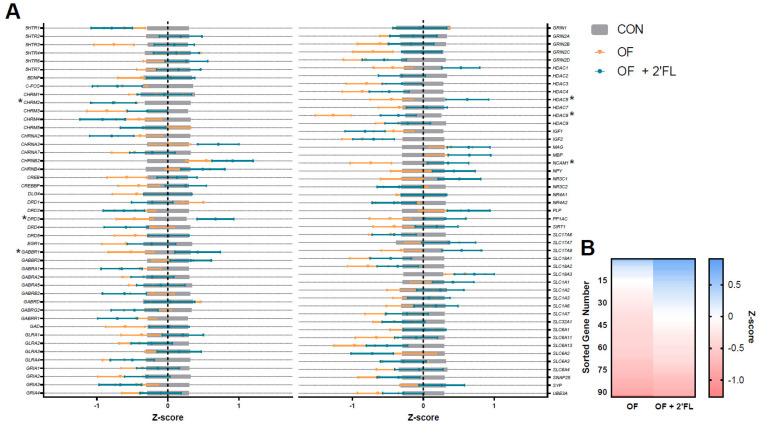
Hippocampal tissue was assessed for the mRNA expression of 93 genes. (**A**) Figure depicts standardized data (mean = 0, standard deviation = 1) centered by control group. Values below zero indicate decreased expression compared to control, whereas values above zero indicate increased expression. Bars show the mean + standard error, and genes significantly impacted by diet are denoted by an asterisk. Accession numbers for each gene can be found in Supplemental Table S7. (**B**) Genes were sorted in descending order by Z-score for each diet, visualizing the abundance of downregulated gene products in the OF group compared to the OF + 2′-FL group. Abbreviations: CON, control group; OF, pigs fed oligofructose; OF + 2′-FL, pigs fed oligofructose and 2′ fucosyllactose.

No samples were found outside the normalization factor ranges for positive controls (0.3–3.0) or housekeeping genes (0.1–10). Some samples demonstrated mRNA counts of some genes below the threshold for background subtraction, and these are detailed in Supplemental Table S7. However, none of the below genes expressed below threshold were found to be affected by the diet. The OF and OF + 2′-FL groups largely had opposite effects compared to the CON group. Hippocampal mRNA expression of the dopamine receptor D3 (DRD3), the GABA type B receptor subunit 1 (GABBR1), the histone deacetylases 5 and 8 (HDAC5/8), the neural cell adhesion molecule 1 (NCAM1), and the cholinergic receptor muscarinic 2 (CHRM2) were all downregulated in the OF group (all *p* < 0.045, Table 3, Figure 6A). Except for HDAC8 and CHRM2, these same genes were upregulated in the OF + 2′-FL group compared to controls (all *p* < 0.045). To visualize the general trend for mRNA to be up- or downregulated as compared to controls, within diet, genes were ordered by descending average Z-score and plotted on a heatmap (Figure 6B). All results can be found in Supplemental Table S7.

### 3.4. Correlation and Linear Regression

**Figure 7:**
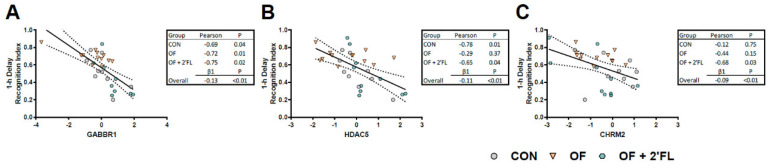
Significant correlations by diet or study-wide linear regression between the recognition index after a 1 h delay and genes affected by diet. No significant relationships were found with the recognition index after a 48 h delay. (**A**) A negative correlation for all diets was found between GABBR1 and the recognition index. Linear regression demonstrated the presence of a significant relationship—for every increase in 1 standard deviation of GABBR1 expression, the recognition index decreased by 0.13. (**B**,**C**) Some dietary groups showed a correlation between the recognition index and either HDAC5 or CHRM2. Overall, linear regression revealed significant negative relationships between mRNA expression and the recognition index. Abbreviations: *GABBR1,* GABA type B receptor subunit 1; *HDAC5*, histone deacetylase 5; *CHRM2,* cholinergic receptor muscarinic 2; CON, control group; OF, pigs fed oligofructose; OF + 2′-FL, pigs fed oligofructose and 2′ fucosyllactose.

Linear regression (independent of diet) and correlations (by diet group) were conducted between only those variables significantly affected by the diet. Although hippocampal tissue, MRI, and behavioral data were collected on separate days, there were significant study-wide relationships between the recognition index after a 1 h delay and CHRM2 (β1 = −0.08, *p* = 0.01), GABBR1 (β1 = −0.13, *p* < 0.01), and HDAC5 (β1 = −0.11, *p* < 0.01) expression. When correlations were assessed by diet, these relationships appeared to be driven by specific dietary groups (Figure 7, Supplemental Table S8). No relationships were observed between gene expression data and the recognition index after a 48 h delay (all *p* > 0.10).

## 4. Discussion

The objective of this study was to assess the effectiveness of OF alone or in combination with 2′-FL at altering recognition memory, brain structure, and hippocampal gene expression. Dietary intake of OF alone produced effects differential to that of OF + 2′-FL concerning recognition memory and hippocampal gene expression, yet both increased the relative volume of the olfactory bulbs compared to controls. An important limitation of the experimental design is the use of different doses for each source of oligosaccharide and lack of a full two-way ANOVA design, thus confounding the ability to delineate between dose and source effects. This choice was made to replicate the doses used from previous clinical trials, however [11,31,32,33]. Here, the analyzed concentration of OF in each group was 3.41–3.62 g/L, and 2′ FL was included in the combination at 1.12 g/L. This is in comparison to previous clinical trials investigating OF in infant formula at ranges from 3 to 5 g/L [31,32] and 2′ FL at ranges from 0.2 to 1.0 g/L [11,33]. Thus, the aim was to assess if there is additional benefit to the addition of 2′ FL in a formula already containing OF, using historically relevant doses for each OS. Still, this study adds to a growing body of literature suggesting that OS can alter behavior and neurobiology. However, the mechanisms of action remain unclear. While many OS have been cited as having such effects [44], it is becoming clear that not all OS act similarly or with the same efficacy.

We chose to use the NOR task using two different delays to assess short-to-intermediate (1 h delay) and long-term (48 h delay) object recognition memory. While CON pigs failed to exhibit recognition memory after both the 1 and 48 h delays, pigs fed OF exhibited recognition memory after a 1 h delay, whereas pigs fed OF + 2′-FL exhibited recognition memory after a 48 h delay. Curiously, pigs fed the combination did not show an improvement in recognition memory after a 1 h delay, demonstrating specificity of the combination to improve type-specific recognition memory. This is potentially due to the requirement of the perirhinal cortex for short- but not long-term memory, and the role of the hippocampus in only long-term recognition memory [45]. As speculated further below, this may also be related to the differences in molecular pathways used for short- and long-term memory.

Pigs fed OF displayed greater number of visits and quicker habituation to the novel object after a 1 h delay, whereas those fed OF + 2′-FL did not habituate to the sample object, but rather maintained a high rate of exploration throughout the trial. Aside from recognition memory, all groups behaved similarly (e.g., total distance moved, frequency, total duration, and mean length of object visits) after a 48 h delay. Though it may appear concerning that the control group was unable to complete the task, we have previously reported a similar phenomenon wherein pigs fed a diet without prebiotics (polydextrose and galactooligosaccharide) could not demonstrate recognition memory [21]. In a follow-up study where the control diet was then supplemented with those same prebiotics and the test diet supplemented further with sialyllactose, no differences in behavioral performance were observed [39]. Given that recognition memory is measured behaviorally in a binary manner (presence or absence thereof), if the goal is to demonstrate a cognitive promoting effect of a nutrient, the use of a control group that is unable to complete the task is necessary to detect subtle improvements in recognition memory.

Where diet had a significant impact on behavioral outcomes, it had no impact on MRS or DTI outcomes, and of the 22 brain regions investigated, only the relative size of the olfactory bulbs was affected. In humans aged 1–17 years of age, absolute volume of the olfactory bulb increases with age, whereas the relative volume decreases continuously starting the first year of age [46]. During this time, olfactory bulb function was correlated with olfactory bulb volume, with increasing volume correlated with increasing function [46,47]. Animals studies have also found that olfactory deprivation results in reduction in olfactory bulb size in opposums [48] and vascular density in rats [49], demonstrating a strong link between size and function. However, we found no relationship between recognition memory and relative olfactory bulb volume (Supplemental Table S8), but a true test of olfactory function would be required to relate olfactory size and function in the pig.

Although the change in relative volume of the olfactory bulb was the only statistically significant outcome, a statistical trend for a change in volume of several brain regions warrants investigation. It is notable that all brain regions affected by the diet were subcortical. The absolute volumes of the caudate, internal capsule, and thalamus and the relative volumes of the caudate, cerebrospinal fluid, and both hippocampi were sensitive to diet. Although the volumes (absolute or relative) of the olfactory bulbs, caudate, and internal capsule are similar between OF and OF + 2′-FL groups, in the latter group, there was a trend for the relative volumes of the left and right hippocampi to be smaller compared to CON and OF groups. This further supports the emerging and consistent pattern where several measures (behavior, structural, or genetic) were divergently affected between OF and OF + 2′-FL groups. Hippocampal function has been traditionally associated with behavioral tasks requiring integration of spatial cues or retention of information over a long period of time. In regard to the novel object recognition task, a study in mice has shown that hippocampal lesion only impaired novel object recognition with a delay of 24 h but not with a delay of 5 min [50]. It is only when recognition memory contains a spatial component (such as the context or location of a stimulus) or long delay that the hippocampus is required, otherwise recognition of “what” was seen requires the perirhinal cortex [45]. It is therefore surprising to observe in the OF + 2′-FL group a trend toward a reduction in relative hippocampi volume (0.05 ≤ *p* < 0.1) concurrent with increased performance in the novel object recognition with a long delay (48 h). Here, the absolute volumes of the hippocampi were similar between groups. However, the relative decrease in volume in the OF + 2′-FL group may suggest a shift in the process of synaptogenesis and/or myelination. Conversely, given the stability between groups in absolute volume, it is possible that the reduction in relative volume of the hippocampi is an artifact of more significant growth in other brain regions.

Of the genes affected by diet in the present study, it appeared that pigs fed OF displayed opposite effects of those fed OF + 2′-FL, and this is evident in the pattern shown in Figure 6B. Overall, pigs fed OF demonstrated greater hippocampal downregulation as compared to controls than pigs fed OF + 2′-FL. Specifically, pigs fed OF displayed reduced gene expression of *DRD3*, *GABBR1*, *HDAC5/8*, *NCAM1*, and *CHRM2* relative to controls. Pigs fed OF + 2′-FL displayed increases in all of the previous genes except for *HDAC8* and *CHRM2*. Although the magnitude of expression was similar for *CHRM2*, a gene known to be related to cognition in humans [51], the downregulation of *HDAC5* was greater for pigs fed OF than those fed OF + 2′-FL. Such a pattern may be related to the apparent difference in behavior observed in the NOR task, but it remains difficult to reconcile the differential effects of OF and OF + 2′-FL on gene expression given their apparent benefit to recognition memory.

Our results share some overlap with previous work examining GOS and FOS on cognition and gene expression. Oral gavage with 3 g/kg FOS or 4 g/kg GOS for 5 weeks has been shown to differentially alter BDNF, NMDAR, and plasma D-serine in adult male rats [5]. BDNF and the glutamatergic NMDA receptor NR1 were greater in the hippocampus of those fed FOS, whereas NR1 was greater in the frontal cortex and NR2 greater in the hippocampus of those fed GOS. Interestingly, we did not see an increase in mRNA expression of *BDNF*, any of the glutamate ionotropic receptor NMDA type subunits (*GRIN1*, *GRIN2A/B/C/D*), or α-amino-3-hydroxy-5-methyl-4-isoxazolepropionic acid (AMPA) type subunits (*GRIA1–4*). We noted similar departures from rodent work in a previous study [21], and these differences may be accounted for by a myriad of differences such as outcome (protein or mRNA expression), animal model (pig or rodent), and the relative difference in development over the course of a four week period between pigs and rodents.

We assessed the relationship between the genes affected by diet and behavioral outcomes. Of the affected genes, *CHRM2*, *GABBR1*, and *HDAC5* were inversely correlated with the recognition index after a 1 h delay. While significant relationships were observed overall, these appeared to be driven by specific dietary groups. The relationship between the recognition index and *CHRM2* was strongest in the OF + 2′-FL group, whereas the relationship between *HDAC5* and the recognition index was driven by the CON and OF + 2′-FL group. All dietary groups showed an inverse relationship between *GABBR1* and the recognition index, and linear regression revealed the strongest and most significant relationship between these two outcomes. None of the affected genes were related to the recognition index after a 48 h delay, suggesting a different mechanism for the behavioral demonstration of recognition memory after intermediate and long delays, and that these mechanisms are sensitive and differentially altered by oligosaccharide supplementation.

The idea that some genes are differentially expressed in context of time or familiarity/novelty of a stimuli is not new [52,53]. However, the possibility that dietary oligosaccharides may differentially alter such processes is novel. The divergence in performance on the NOR task and *GABBR1* expression (and many other genes) by the OF and OF + 2′-FL groups together with the relationship between the recognition index and *GABBR1* expression highlight a potential mechanistic link connecting the two phenomena. To our knowledge, there has been little investigation into the connection between prebiotic intake and GABAergic processes. In context of probiotics, BALB/c mice orally gavaged with broth containing *L. rhamnosus* (JB-1) for 28 d displayed greater movement in an open field test, less time immobile during a forced swim test, greater entries to the open arm in an elevated plus maze, and increased memory on a fear conditioning task; indices demonstrating reduced response to stress and improved memory [54]. These changes were simultaneous with reduced mRNA expression of the *GABA_B1b_* receptor in the amygdala, locus coeruleus, and hippocampus, and increased expression in the cingulate 1 and prelimbic cortices. A follow-up study using magnetic resonance spectroscopy in mice provided JB-1 demonstrated increased brain GABA after 4 weeks of consumption [55]. For object recognition, it appears that hippocampal reductions in GABA_B_ receptor expression may be beneficial. Cavallaro et al. [52] proposed that downregulation of hippocampal GABA_B_ receptor signaling may improve short term memory. In support of this hypothesis, Baclofen, a GABA_B_ receptor agonist, has been shown to dose-dependently impair acquisition and storage of object recognition memory, whereas GABA_B_ receptor antagonism can prevent baclofen-induced impairments [56]. Although the effects of GABA_B_ agonism and antagonism vary by dose, route, and behavioral task used (for review see Heaney and Kinney [57]), it appears that both oligosaccharides and probiotics [54,55] are linked to beneficial alteration of GABA receptor expression in the brain.

While this study confirms several reports showing that various OS improve behavioral performance in both human and animal models [6,13,14,21,22,23,58], it is one of the first to examine the potential of human and non-human milk OS together. Adult mice and rats fed chow containing 0.312% or 0.625% 2′-FL for 12 or 5 weeks, respectively, showed increased and longer-lasting potentiation of Schaffer collateral neurons in the CA1 region of the hippocampus [13]. Supplemented mice displayed increased performance on place learning, working memory, and fixed-ratio lever-pressing tasks in an operant box, suggesting that 2′-FL supplementation enhanced multiple cognitive domains. In a follow-up study by the same group, subdiaphragmatic bilateral vagotomy was used to assess the requirement of the vagus nerve for 2′-FL-mediated increases in cognition in rodents [14]. Vagotomy abolished 2′-FL-mediated increases in hippocampal long-term potentiation. However, all groups (sham/vagotomy and control/2′-FL) were still able to perform above criterion in a fixed-ratio lever pressing task. By the end of training, 2′-FL/sham animals displayed greater lever presses than 2′-FL/vagotomy or control/vagotomy animals, indicating that while not required for behavioral performance, vagal communication is necessary for 2′-FL-induced increases. Together, these data suggest that the vagus nerve has a crucial role in mediating gut–brain-related increases in cognitive ability.

Given the significant evidence described above demonstrating preliminary but growing data suggesting that prebiotics of many sources can improve cognition, there are few compelling arguments without speculation upon why. This extends to the present research, wherein alterations in brain volume and gene expression are not sufficient to describe the behavioral effects shown. Ultimately, the field of study is too young to make reasonable inferences on why prebiotics can improve cognition. These limitations add to the field’s novelty and provide compelling evidence for why future research is highly warranted.

## 5. Conclusions

We investigated the impact of early life dietary supplementation with OF alone or in combination with 2′-FL on cognition, structural brain development, and hippocampal gene expression. Feeding either OS had little impact on brain structure. However, they had differential effects on cognition and hippocampal gene expression. We found relationships between novel object performance and mRNA expression of genes related to GABAergic, cholinergic, and histone deacetylation processes. Overall, these data highlight the potential for these two OS to be used in early life nutrition to promote cognition, and the potential interactions between OS of human and non-human sources. However, more research on the underlying mechanisms is warranted.

## Figures and Tables

**Table 1:** Formulated nutrient composition of the base formula ^1,2^.

Nutrient	Units	Base Formula		Final Composition ^3^	
		Per kg	Per Liter	Per kg	Per Liter
**Energy and Macronutrients**					
Metabolizable Energy	kcal	4286.4	857.3	3989.4	797.9
Crude Protein	g	241.0	48.2	224.3	44.9
Crude Fat	g	241.0	48.2	224.3	44.9
Lactose	g	369.1	73.8	343.5	68.7
Crude Fiber	mg	20.0	4.0	18.6	3.7
Ash	g	85.3	17.1	79.4	15.9
**Minerals**					
Calcium	mg	1000.0	200.0	930.7	186.1
Copper	mg	12.1	2.4	11.2	2.2
Total Phosphorous	mg	800.0	160.0	744.6	148.9
Potassium	mg	1835.0	367.0	1707.8	341.6
Selenium	μg	875.0	175.0	814.4	162.9
Sodium	mg	345.0	69.0	321.1	64.2
Zinc	mg	120.0	24.0	111.7	22.3
**Vitamins and Other Nutrients**					
Vitamin A	IU	82,427.3	16,485.5	76,715.0	15,343.0
Vitamin D	IU	11,563.9	2312.8	10,762.5	2152.5
Vitamin E	IU	253.3	50.7	235.7	47.1
Lysine	g	25.2	5.0	23.4	4.7
Methionine + Cysteine	g	9.9	2.0	9.2	1.8

^1^ ProNurse^®^ Specialty Milk Replacer (Purina Animal Nutrition, Gray Summit, MO, USA) was used as the base formula and nutritional composition is adapted from advertised nutrient composition. ^2^ Milk was reconstituted at a rate of 200 g of milk replacer per 800 g of water. ^3^ The base formula was diluted 6.93% to allow addition of oligosaccharides.

**Table 2:** Carbohydrate content of the diets ^1^.

	Oligofructose ^2^		2′-FL ^3^		Additional Lactose		Total OS		Total Lactose		Total Carbohydrate	
Diet, g	Per kg	Per Liter	Per kg	Per Liter	Per kg	Per Liter	Per kg	Per Liter	Per kg	Per Liter	Per kg	Per Liter
Formulated												
CON	0.00	0.00	0.00	0.00	69.30	13.86	0.00	0.00	412.79	82.56	412.79	82.56
OF	26.08	5.22	0.00	0.00	61.98	8.64	26.08	5.22	386.71	77.34	412.79	82.56
OF + 2′-FL	26.08	5.22	4.92	0.98	38.30	7.66	31.00	6.20	381.79	76.36	412.79	82.56
Analyzed												
CON	0.00	0.00	0.00	0.00	NQ	NQ	0.00	0.00	NQ	NQ	NQ	NQ
OF	18.10	3.62	0.00	0.00	NQ	NQ	18.10	3.62	NQ	NQ	NQ	NQ
OF + 2′-FL	17.05	3.41	5.60	1.12	NQ	NQ	22.65	4.53	NQ	NQ	NQ	NQ

^1^ Abbreviations: 2′-FL, 2′-OS, oligosaccharide; CON, control group; HMO, pigs fed human milk oligosaccharides; BMOS; pigs fed bovine milk oligosaccharides, BMOS + HMO, pigs fed both human and bovine milk oligosaccharides; NQ, not quantified. ^2^ Orafti^®^ P95; Beneo-Orafti, Tienen, Belgium. ^3^ Glycom, Hørsholm, Denmark.

**Table 3:** Standardized RNA expression ^1^.

	CON		OF		OF + 2′-FL			
Measure ^2^	N	Mean	N	Mean	N	Mean	SEM	*p*-Value ^3^
*CHRM2* ^4^	12	0 ^a^	12	−0.75 ^a^	12	−0.77 ^a^	0.32	0.045
*DRD3*	12	0 ^ab^	12	−0.47 ^b^	12	0.67 ^a^	0.26	0.016
*GABBR1*	12	0 ^ab^	12	−0.52 ^b^	12	0.42 ^a^	0.32	0.037
*HDAC5*	11	0 ^ab^	12	−0.45 ^b^	12	0.62 ^a^	0.30	0.012
*HDAC8*	12	0 ^a^	12	−1.27 ^b^	12	−0.35 ^a^	0.25	0.003
*NCAM1*	12	0 ^a,b^	12	−0.74 ^b^	12	0.35 ^a^	0.29	0.011

^1^ Abbreviations: CON, control group; OF, pigs fed oligofructose; OF + 2′-FL, pigs fed oligofructose and 2′-fucosyllactose; SEM, standard error of the mean; CHRM2, cholinergic receptor muscarinic 2; DRD3, dopamine receptor D3; GABBR1, GABA type B receptor subunit 1; HDAC5/8, histone deacetylases 5 and 8; NCAM1, neural cell adhesion molecule 1. ^2^ Standardized values for mRNA expression centered by control group. Only measures significantly altered by diet are shown. ^3^ Data analyzed via one-way ANOVA with post-hoc Tukey adjustment for mean separation. ^4^ Mean separation insignificant after Tukey adjustment. ^a,b,c^ Mean superscripts without a common letter differ (*p* < 0.05).

## Supplementary Materials

The following are available online at https://www.mdpi.com/2072-6643/12/7/2131/s1, Table S1. Exploratory behavior during the novel object recognition task ^1,2^. Table S2. Absolute Brain Volume ^1,2^. Table S3. Relative Brain Volume ^1,2^. Table S4. Diffusion Tensor Imaging ^1,2^. Table S5. Fractional Anisotropy ^1,2^. Table S6. Magnetic Resonance Spectroscopy ^1,2^. Table S7. Gene Expression ^1^. Table S8. Correlations between the recognition index and significant MRI and gene expression outcomes ^1,2^.
