# Supplementary material for: Dietary Oligofructose Alone or in Combination with 2′-Fucosyllactose Differentially Improves Recognition Memory and Hippocampal mRNA Expression
Source: Nutrients. 2020 Jul 17;12(7):2131. doi: 10.3390/nu12072131 (PMC7400822; doi:10.3390/nu12072131)
Supplement: Supplementary file 1 [file nutrients-12-02131-s001.pdf]

Dietary oligofructose alone or in combination with 2'fucosyllactose differentially improves recognition memory and alters brain structure and hippocampal mRNA expression

Stephen A. Fleming[1,2], Austin T. Mudd[1,2], Jonas Hauser[3], Jian Yan[4], Sylviane Metairon[3], Pascal Steiner[3], Sharon M. Donovan[5,6], Ryan N. Dilger[1,2,5,6*]

[1]Piglet Nutrition and Cognition Laboratory, Department of Animal Sciences, University of Illinois, Urbana, IL, USA

[2]Neuroscience Program, University of Illinois, Urbana, Illinois, USA
[3]Nestlé Research, Lausanne, Switzerland
[4]Nestlé Product Technology Center Nutrition, Vevey, Switzerland
[5]Department of Food Science and Human Nutrition, University of Illinois, Urbana, IL, USA
[6]Division of Nutritional Sciences, University of Illinois, Urbana, Illinois, USA

*Correspondence:
Dr. Ryan N. Dilger
University of Illinois
Department of Animal Sciences
Piglet Nutrition and Cognition Laboratory
1207 W. Gregory Street
186 Animal Sciences Laboratory
Urbana, IL, 61801, USA
(217) 333-2006
rdilger2@illinois.edu

Supplemental Table 1. Exploratory behavior during the novel object recognition task[1,2]

	Diet							
	CON		OF		OF + 2′FL		Pooled	
**Measure**	**N**	**Mean**	**N**	**Mean**	**N**	**Mean**	**SEM**	***P*-value**[3]
Habituation 1								
Total distance moved, m	12	119.48	12	104.13	12	102.27	8.99	0.208
Δ distance moved, m/min	12	2.04	12	0.88	12	1.47	0.35	0.084
Time spent in the center of the arena, %	12	32.91	12	33.02	12	32.62	2.66	0.991
Habituation 2								
Total distance moved, m	12	81.12	12	80.98	11	81.71	10.15	0.998
Δ distance moved, m/min	12	0.30	12	0.50	11	0.19	0.26	0.572
Time spent in the center of the arena, %	12	42.37	12	31.31	11	38.99	4.66	0.138
Sample Phase								
Total object visit time, s	12	77.52	12	82.94	12	88.10	15.51	0.889
Number of object visits	12	11.88	12	13.50	12	12.38	1.65	0.751
Mean object visit time, s	12	6.72	12	6.86	11	6.99	1.25	0.985
Latency to first object visit, s	11	21.29	12	8.86	11	21.07	8.73	0.504
Habituation towards both objects, s/min	12	-3.42	12	-5.11	11	-3.69	1.21	0.550
Total distance moved, m	12	32.08	12	36.98	12	36.65	3.63	0.574
Δ distance moved, m/min	12	0.41	12	0.59	12	0.60	0.24	0.788
Time spent in the center of the arena, %	12	58.31	12	53.57	12	54.99	5.08	0.796
Test Phase: 1-hour delay								
Novel object visit time, s	10	44.58	12	72.52	10	45.62	12.39	0.140
Number of novel object visits	11	4.29	12	6.75	11	4.20	0.76	0.022
Mean novel object visit time, s	11	11.86	12	11.73	11	8.20	2.50	0.501
Latency to first novel object visit, s	9	27.78	12	13.96	11	55.01	15.46	0.108
Habituation to the novel object, s/min	10	-1.32	12	-6.68	11	-3.42	2.03	0.045
Sample object visit time, s	10	39.90	12	32.81	10	39.65	12.14	0.872
Number of sample object visits	11	5.59	12	5.75	12	5.75	0.94	0.989
Mean sample object visit time, s	11	6.48	12	6.91	11	5.33	1.75	0.749
Latency to first sample object visit, s	10	21.62	12	22.87	10	32.27	10.80	0.746
Habituation to the sample object, s/min	10	-2.25	12	-1.52	10	0.70	0.91	0.038
Total object visit time, s	11	83.64	12	105.33	12	111.93	23.23	0.642
Number of object visits	11	9.00	12	12.50	12	10.75	1.55	0.278
Mean object visit time, s	11	8.38	12	9.43	11	7.64	2.15	0.811
Latency to first object visit, s	11	13.09	12	7.57	11	29.86	9.84	0.258
Habituation towards both objects, s/min	11	-3.41	12	-8.20	12	-3.62	1.75	0.056
Total distance moved, m	11	29.49	11	28.95	12	32.32	3.41	0.745
Δ distance moved, m/min	11	0.16	12	0.29	12	0.23	0.27	0.937
Time spent in the center of the arena, %	11	61.17	12	61.34	12	60.95	6.03	0.999

[1]Abbreviations: CON, control group; OF, pigs fed oligofructose; OF + 2′FL, pigs fed oligofructose and 2′fucosyllactose; SEM, standard error of the mean; m, meter; Δ, change in; min, minute; %, percent; s, second.
[2]Data presented are least square means.
[3]*P*-values derived from mixed model ANOVA.

Supplemental Table 1. Continued[1,2]

	Diet							
	CON		OF		OF + 2′FL		Pooled	
**Measure**	**N**	**Mean**	**N**	**Mean**	**N**	**Mean**	**SEM**	***P*-value**[3]
Test Phase: 48-hour delay								
Novel object visit time, s	10	37.16	12	46.94	11	68.87	10.67	0.107
Number of novel object visits	11	5.18	12	6.08	12	8.08	0.90	0.073
Mean novel object visit time, s	11	7.37	11	8.28	12	8.70	1.56	0.822
Latency to first novel object visit, s	10	23.29	11	24.85	11	23.80	8.99	0.992
Habituation to the novel object, s/min	10	-2.55	12	-1.91	11	-3.96	1.25	0.465
Sample object visit time, s	11	33.57	12	31.65	11	29.70	8.26	0.930
Number of sample object visits	11	5.45	12	5.33	11	4.73	0.73	0.755
Mean sample object visit time, s	11	5.91	12	5.70	12	5.71	1.15	0.984
Latency to first sample object visit, s	11	42.44	12	34.47	11	46.74	16.14	0.856
Habituation to the sample object, s/min	10	0.31	11	-0.88	11	-0.90	0.72	0.402
Total object visit time, s	11	70.77	12	91.02	12	98.57	17.41	0.491
Number of object visits	11	9.75	12	11.42	12	12.42	1.44	0.427
Mean object visit time, s	11	6.32	12	7.24	11	7.67	1.04	0.594
Latency to first object visit, s	10	9.14	12	24.22	12	19.25	8.96	0.477
Habituation towards both objects, s/min	10	-2.24	12	-1.94	11	-4.86	1.48	0.289
Total distance moved, m	11	33.19	12	32.61	12	34.08	3.62	0.954
Δ distance moved, m/min	11	-0.34	12	0.14	12	0.56	0.27	0.079
Time spent in the center of the arena, %	11	61.62	12	59.13	11	64.00	5.10	0.790

[1]Abbreviations: CON, control group; OF, pigs fed oligofructose; OF + 2′FL, pigs fed oligofructose and 2′fucosyllactose; SEM, standard error of the mean; s, second; m, meter; min, minute; %, percent; Δ, change in.
[2]Data presented are least square means.
[3]*P*-values derived from mixed model ANOVA.

Supplemental Table 2. Absolute Brain Volume[1,2]

	Diet							
	CON		OF		OF + 2′FL			
Brain Region, mm[3]	N	Mean	N	Mean	N	Mean	Pooled SEM	*P*-Value[3]
Caudate	11	501.7	12	482.6	11	476.2	7.7	0.069
Cerebellum	11	6064.1	12	5983.1	11	6022.8	90.5	0.811
Cerebral Aqueduct	11	46.6	12	47.0	11	46.8	1.7	0.991
Corpus Callosum	11	364.4	12	363.4	11	362.7	7.8	0.976
Cerebrospinal Fluid	11	5262.7	12	6163.4	11	6919.1	581.3	0.102
Fourth Ventricle	10	48.8	12	48.4	11	51.2	1.6	0.322
Grey Matter	11	37999.0	12	37168.7	11	37141.8	533.1	0.429
Hypothalamus	11	249.7	12	240.4	11	242.4	3.4	0.135
Internal Capsule	10	1491.7	12	1494.5	11	1543.5	26.1	0.097
Lateral Ventricle	11	487.0	12	517.8	11	490.7	21.4	0.444
Left Cortex	11	16531.7	12	16488.2	11	16503.9	258.4	0.985
Left Hippocampus	11	479.9	12	479.4	11	465.3	7.4	0.232
Medulla	11	1816.8	12	1815.0	11	1832.0	33.0	0.872
Midbrain	11	1869.8	12	1860.9	11	1875.6	27.9	0.918
Olfactory Bulb	11	2238.0	12	2383.3	11	2391.8	57.5	0.051
Pons	11	1072.9	12	1073.1	11	1102.4	22.6	0.457
Putamen-Globus Pallidus	11	412.0	12	403.3	11	401.1	7.4	0.379
Right Cortex	11	17065.1	12	17037.6	11	17177.4	258.9	0.835
Right Hippocampus	11	491.7	12	489.0	11	477.0	7.1	0.220
Thalamus	11	1624.5	12	1571.7	11	1598.6	19.9	0.086
Third Ventricle	11	47.6	12	48.7	11	50.7	2.3	0.634
White Matter	11	16926.4	12	17128.5	11	17338.6	395.0	0.616
Whole Brain	11	66353.8	12	65646.6	11	66239.5	1063.4	0.766

[1]Abbreviations: mm, millimeter; CON, control group; OF, pigs fed oligofructose; OF + 2′FL, pigs fed oligofructose and 2′fucosyllactose; SEM, standard error of the mean.

[2]Data presented are least square means.

[3]*P*-values derived from mixed model ANOVA.

Supplemental Table 3. Relative Brain Volume[1,2]

Brain Region, %TBV	Diet						Pooled SEM	*P*-Value[3]
	CON		OF		OF + 2′FL			
	N	Mean	N	Mean	N	Mean		
Caudate	11	0.759	12	0.736	11	0.719	0.016	0.065
Cerebellum	11	9.131	12	9.119	11	9.113	0.117	0.992
Cerebral Aqueduct	11	0.070	12	0.072	11	0.071	0.002	0.906
Corpus Callosum	11	0.550	12	0.554	11	0.548	0.013	0.869
Cerebrospinal Fluid	11	7.911	12	9.351	11	10.459	0.806	0.090
Fourth Ventricle	10	0.074	12	0.074	11	0.077	0.002	0.408
Grey Matter	11	57.210	12	56.639	11	56.202	0.719	0.616
Hypothalamus	11	0.376	12	0.367	11	0.367	0.006	0.337
Internal Capsule	11	2.283	12	2.277	11	2.331	0.039	0.399
Lateral Ventricle	11	0.736	12	0.790	11	0.742	0.036	0.364
Left Cortex	11	24.929	12	25.114	11	24.929	0.228	0.752
Left Hippocampus	11	0.723	12	0.731	11	0.704	0.010	0.063
Medulla	11	2.746	12	2.766	11	2.769	0.056	0.893
Midbrain	11	2.817	12	2.836	11	2.837	0.038	0.902
Olfactory Bulb	11	3.379	12	3.628	11	3.617	0.068	0.019
Pons	11	1.615	12	1.635	11	1.665	0.022	0.273
Putamen-Globus Pallidus	11	0.622	12	0.615	11	0.606	0.011	0.395
Right Cortex	11	25.745	12	25.957	11	25.952	0.265	0.730
Right Hippocampus	11	0.741	12	0.745	11	0.721	0.008	0.056
Thalamus	11	2.450	12	2.397	11	2.417	0.035	0.219
Third Ventricle	11	0.071	12	0.074	11	0.077	0.003	0.529
White Matter	11	25.543	12	26.099	11	26.209	0.586	0.468

[1]Abbreviations: %TBV, percent total brain volume; CON, control group; OF, pigs fed oligofructose; OF + 2′FL, pigs fed oligofructose and 2′fucosyllactose; SEM, standard error of the mean.

[2]Data presented are least square means.

[3]*P*-values derived from mixed model ANOVA.

Supplemental Table 4. Diffusion Tensor Imaging[1,2]

	Diet							
	CON		OF		OF + 2′FL			
**Measure, x10^{-3} mm^2/s**	**N**	**Mean**	**N**	**Mean**	**N**	**Mean**	**Pooled SEM**	***P*-Value**[3]
Axial diffusivity								
Caudate	11	1.188	10	1.219	10	1.237	0.019	0.193
Corpus Callosum	11	1.614	11	1.665	10	1.681	0.066	0.713
Cerebellum	11	1.347	11	1.345	10	1.392	0.029	0.424
Left Hippocampus	11	1.517	10	1.448	10	1.479	0.037	0.359
Right Hippocampus	11	1.499	11	1.489	10	1.505	0.050	0.971
Internal Capsule	11	1.276	11	1.275	10	1.282	0.009	0.751
Left Cortex	11	1.364	11	1.361	10	1.352	0.010	0.591
Right Cortex	11	1.403	11	1.407	10	1.418	0.016	0.795
Thalamus	11	1.177	11	1.178	10	1.169	0.014	0.788
DTI-derived white matter	11	1.406	11	1.403	10	1.400	0.012	0.903
Atlas-derived white matter	11	1.459	11	1.447	10	1.450	0.016	0.727
Radial diffusivity								
Caudate	11	0.706	10	0.731	10	0.736	0.012	0.187
Corpus Callosum	11	1.020	11	1.057	10	1.076	0.042	0.590
Cerebellum	11	1.054	11	1.045	10	1.088	0.026	0.470
Left Hippocampus	10	0.945	11	0.920	10	0.926	0.036	0.809
Right Hippocampus	11	0.940	11	0.934	10	0.965	0.037	0.818
Internal Capsule	10	0.659	11	0.654	10	0.647	0.005	0.207
Left Cortex	11	0.850	11	0.843	10	0.839	0.008	0.584
Right Cortex	11	0.861	11	0.863	10	0.866	0.012	0.939
Thalamus	11	0.720	11	0.720	10	0.732	0.008	0.225
DTI-derived white matter	11	0.879	11	0.872	10	0.872	0.008	0.769
Atlas-derived white matter	11	0.911	11	0.901	10	0.904	0.011	0.643
Mean diffusivity								
Caudate	11	0.867	10	0.893	10	0.903	0.014	0.165
Corpus Callosum	11	1.217	11	1.260	10	1.278	0.050	0.642
Cerebellum	11	1.152	11	1.145	10	1.190	0.027	0.452
Left Hippocampus	10	1.134	10	1.078	10	1.111	0.031	0.364
Right Hippocampus	11	1.127	11	1.119	10	1.145	0.041	0.895
Internal Capsule	10	0.866	11	0.861	10	0.859	0.005	0.521
Left Cortex	11	1.021	11	1.016	10	1.010	0.009	0.581
Right Cortex	11	1.042	11	1.044	10	1.050	0.013	0.882
Thalamus	10	0.875	11	0.873	10	0.878	0.009	0.778
DTI-derived white matter	11	1.055	11	1.049	10	1.048	0.009	0.820
Atlas-derived white matter	11	1.093	11	1.083	10	1.086	0.012	0.676

[1]Abbreviations: mm, millimeter; s, second; CON, control group; OF, pigs fed oligofructose; OF + 2′FL, pigs fed oligofructose and 2′fucosyllactose; SEM, standard error of the mean; DTI, diffusion tensor imaging.

[2]Data presented are least square means.

[3]*P*-values derived from mixed model ANOVA.

Supplemental Table 5. Fractional Anisotropy[1,2]

	Diet							
	CON		OF		OF + 2'FL			
**Brain Region, arbitrary units**	**N**	**Mean**	**N**	**MeaN**	**N**	**Mean**	**Pooled SEM**	***P*-Value[3]**
Caudate	10	0.339	11	0.330	10	0.333	0.005	0.387
Corpus Callosum	11	0.296	11	0.293	10	0.291	0.005	0.709
Cerebellum	10	0.167	11	0.168	10	0.169	0.004	0.940
Left Hippocampus	9	0.301	11	0.308	10	0.301	0.006	0.492
Right Hippocampus	11	0.297	11	0.297	10	0.285	0.006	0.336
Internal Capsule	10	0.409	11	0.412	10	0.420	0.004	0.173
Left Cortex	10	0.315	11	0.316	10	0.315	0.001	0.801
Right Cortex	10	0.329	11	0.324	10	0.328	0.002	0.259
Thalamus	11	0.313	11	0.320	10	0.306	0.006	0.245
DTI-derived white matter	10	0.314	11	0.315	10	0.314	0.001	0.952
Atlas-derived white matter	11	0.314	11	0.315	10	0.315	0.002	0.494

[1]Abbreviations: CON, control group; OF, pigs fed oligofructose; OF + 2'FL, pigs fed oligofructose and 2'fucosyllactose; SEM, standard error of the mean; DTI, diffusion tensor imaging.

[2]Data presented are least square means.

[3]*P*-values derived from mixed model ANOVA.

Supplemental Table 6. Magnetic Resonance Spectroscopy[1,2]

	Diet							
	CON		OF		OF + 2’FL			
**Metabolite, ppm**	**N**	**Mean**	**N**	**Mean**	**N**	**Mean**	**Pooled SEM**	***P*-Value[3]**
Glutathione	9	1.080	9	1.148	12	1.286	0.102	0.303
Myo-inositol	10	4.231	10	4.618	12	4.438	0.282	0.536
N-acetylaspartate	10	4.250	10	4.224	12	4.120	0.176	0.836
γ-amino butyric acid	11	1.396	10	1.393	12	1.366	0.064	0.893

[1]Abbreviations: ppm, parts per million; CON, control group; OF, pigs fed oligofructose; OF + 2’FL, pigs fed oligofructose and 2’fucosyllactose; SEM, standard error of the mean; SEM, standard error of the mean.

[2]Data presented are least square means.

[3]*P*-values derived from mixed model ANOVA.

Supplemental Table 7. Gene Expression[1]

	Diet								
	CON		OF		OF + 2′FL				
**Measure[3]**	**N**	**Mean**	**N**	**Mean**	**N**	**Mean**	**Pooled SEM**	***P*-Value[2]**	**Accession Number**
5HTR1	12	0	12	-0.61	12	-0.79	0.29	0.115	XM_005672476.2
5HTR2	12	0	12	0.18	12	0.18	0.30	0.888	XM_013980517.1
5HTR3[5]	12	0	12	-0.76	12	0.09	0.28	0.070	XM_003357301.3
5HTR4	12	0	12	0.15	12	0.12	0.32	0.919	NM_001001267.1
5HTR6	12	0	12	-0.05	12	0.26	0.30	0.732	XM_003356173.3
5HTR7	12	0	12	-0.13	11	0.15	0.31	0.809	NM_214085.1
BDNF	12	0	11	-0.37	12	0.04	0.34	0.505	XM_005654684.2
C-FOS	12	0	12	-0.64	12	-0.71	0.35	0.065	NM_001123113.1
CHRM1	12	0	12	-0.17	12	-0.05	0.38	0.836	NM_214034.1
CHRM2[4]	12	0[a]	12	-0.75[a]	12	-0.77[a]	0.32	0.045	NM_214261.1
CHRM3	12	0	12	-0.86	12	-0.29	0.28	0.099	NM_001123098.1
CHRM4[5]	12	0	12	-0.41	12	-0.92	0.32	0.054	XM_003122828.4
CHRM5	11	0	12	0.00	12	-0.35	0.32	0.569	XM_013997263.1
CHRNA2	12	0	12	-0.39	12	-0.79	0.31	0.108	XM_003132824.3
CHRNA3[5]	12	0	12	0.03	11	0.71	0.29	0.152	XM_013988980.1
CHRNA7	12	0	12	-0.47	12	-0.22	0.32	0.472	XM_013993241.1
CHRNB2[5]	12	0	12	0.54	12	0.91	0.29	0.090	XM_003125722.3
CHRNB4	12	0	12	0.28	12	0.49	0.31	0.497	XM_013988981.1
CREB	12	0	12	-0.58	11	0.13	0.28	0.184	NM_001099929.1
CREBBP	12	0	12	-0.41	12	0.25	0.29	0.268	XM_003354647.4
DLG4	12	0	12	-0.44	12	0.00	0.34	0.402	XR_001303340.1
DRD1[5]	12	0	12	0.21	12	-0.22	0.29	0.590	XM_005672535.2

[1]Abbreviations: CON, control group; OF, pigs fed oligofructose; OF + 2′FL, pigs fed oligofructose and 2′fucosyllactose; SEM, standard error of the mean.

[2]Data analyzed via two-way ANOVA with post-hoc Tukey adjustment for multiple comparisons.

[3]Standardized values for mRNA expression (mean = 0, standard deviation = 1) centered by control group.

[4]Mean separation insignificant after Tukey adjustment.

[5]Number of samples below threshold (median of negative controls): *5HTR3*, 9: *CHRM4*, 5: *CHRNA3*, 1: *CHRNB2*, 1: *DRD1*, 1: *DRD2*, 3: *DRD4*, 18: *GABBR2*, 1: *GLRA4*, 9: *GRIN2C*, 2: *SLC18A2*, 8: *SLC6A2*, 15: *SLC6A3*, 14: *SLC6A4*, 4.

[abc]Means without a common superscript differ ($P < 0.05$).

Supplemental Table 7. Continued[1]

	Diet								
	CON		OF		OF + 2'FL				
Measure[3]	N	Mean	N	Mean	N	Mean	Pooled SEM	*P*-Value[2]	Accession Number
DRD2[5]	12	0	12	-0.46	12	-0.62	0.29	0.291	XM_005667325.2
DRD3	12	0[ab]	12	-0.47[b]	12	0.67[a]	0.26	0.016	XM_013982425.1
DRD4[5]	12	0	12	-0.22	12	-0.59	0.31	0.343	XM_003122390.2
DRD5	12	0	12	-0.46	12	0.01	0.29	0.425	XM_013989284.1
EGR1	12	0	12	-0.59	11	-0.23	0.34	0.251	XM_003123974.5
GABBR1	12	0[ab]	12	-0.52[b]	12	0.42[a]	0.32	0.037	NM_001123114.1
GABBR2[5]	12	0	12	0.07	12	0.32	0.29	0.722	XM_003122032.5
GABRA1	12	0	12	-0.39	12	-0.65	0.29	0.283	XM_013984876.1
GABRA2	12	0	12	-0.34	12	-0.22	0.30	0.706	XM_013978645.1
GABRA5	12	0	12	-0.21	12	-0.10	0.34	0.845	XM_005654471.2
GABRB2	12	0	12	-0.22	12	-0.61	0.31	0.276	XM_013984878.1
GABRD	12	0	12	0.11	12	0.03	0.35	0.951	XM_013988717.1
GABRG2	12	0	12	-0.29	12	-0.47	0.33	0.450	XM_003359825.3
GABRR1	12	0	12	-0.44	12	-0.71	0.28	0.218	XM_013992686.1
GAD	12	0	12	-0.60	12	0.02	0.28	0.233	NM_213894.1
GLRA1	12	0	12	-0.37	12	0.21	0.29	0.365	XM_013984909.1
GLRA2	12	0	11	-0.40	12	-0.23	0.29	0.637	XM_013985912.1
GLRA3	12	0	12	-0.02	11	0.16	0.31	0.889	XM_003132832.5
GLRA4[5]	12	0	12	-0.61	12	-0.50	0.31	0.266	XM_003135271.3
GRIA1	12	0	12	-0.36	12	-0.14	0.30	0.689	XM_003359841.4
GRIA2	12	0	12	-0.67	12	-0.29	0.32	0.222	XM_005656504.2
GRIA3	12	0	12	-0.44	12	-0.67	0.30	0.232	XM_003135356.4

[1]Abbreviations: CON, control group; OF, pigs fed oligofructose; OF + 2'FL, pigs fed oligofructose and 2'fucosyllactose; SEM, standard error of the mean.

[2]Data analyzed via two-way ANOVA with post-hoc Tukey adjustment for multiple comparisons.

[3]Standardized values for mRNA expression (mean = 0, standard deviation = 1) centered by control group.

[4]Mean separation insignificant after Tukey adjustment.

[5]Number of samples below threshold: *5HTR3*, 9: *CHRM4*, 5: *CHRNA3*, 1: *CHRNB2*, 1: *DRD1*, 1: *DRD2*, 3: *DRD4*, 18: *GABBR2*, 1: *GLRA4*, 9: *GRIN2C*, 2: *SLC18A2*, 8: *SLC6A2*, 15: *SLC6A3*, 14: *SLC6A4*, 4.

[abc]Means without a common superscript differ ($P < 0.05$).

Supplemental Table 7. Continued[1]

	Diet								
	CON		OF		OF + 2′FL				
Measure[3]	N	Mean	N	Mean	N	Mean	Pooled SEM	*P*-Value[2]	Accession Number
GRIA4	12	0	12	-0.36	11	-0.10	0.29	0.668	XM_013979342.1
GRIN1	12	0	12	0.00	12	-0.05	0.38	0.975	XM_013992346.1
GRIN2A	12	0	12	-0.28	11	-0.14	0.33	0.746	XM_003481048.3
GRIN2B	12	0	12	-0.61	11	-0.17	0.32	0.267	XM_003355567.3
GRIN2C[5]	12	0	12	-0.71	12	-0.02	0.28	0.141	XM_013980765.1
GRIN2D	11	0	12	-0.81	12	-0.55	0.31	0.087	XM_003127274.4
HDAC1	11	0	12	-0.43	12	0.53	0.27	0.061	XM_013999116.1
HDAC2	12	0	12	-0.30	11	-0.30	0.33	0.645	XM_001925318.5
HDAC3	12	0	12	-0.80	12	-0.30	0.28	0.143	XM_013995254.1
HDAC4	12	0	12	-0.86	11	-0.48	0.28	0.107	XM_005657593.2
HDAC5	11	0[ab]	12	-0.45[b]	12	0.62[a]	0.30	0.012	XM_013980921.1
HDAC7	12	0	12	-0.34	11	0.05	0.29	0.606	XM_013988449.1
HDAC8	12	0[a]	12	-1.27[b]	12	-0.35[a]	0.25	0.003	XM_013990927.1
HDAC9	12	0	12	-0.35	12	-0.22	0.32	0.658	XM_013979765.1
IGF1	12	0	12	-0.42	12	-0.82	0.28	0.131	NM_214256.1
IGF2	12	0	12	-0.86	12	-0.70	0.29	0.084	NM_213883.2
MAG	12	0	12	0.39	11	0.64	0.30	0.316	XM_013998313.1
MBP	12	0	12	0.35	12	0.65	0.30	0.305	NM_001001546.2
NCAM1	12	0[ab]	12	-0.74[b]	12	0.35[a]	0.29	0.011	XM_005667343.2
NPY	12	0	12	-0.16	12	0.43	0.30	0.360	NM_001256367.1
NR3C1	12	0	12	-0.30	12	0.51	0.30	0.137	NM_001008481.1
NR3C2	12	0	12	-0.25	12	-0.34	0.31	0.674	XM_013978843.1
NR4A1	12	0	12	-0.06	11	0.01	0.32	0.985	FJ548761.1

[1]Abbreviations: CON, control group; OF, pigs fed oligofructose; OF + 2′FL, pigs fed oligofructose and 2′fucosyllactose; SEM, standard error of the mean.
[2]Data analyzed via two-way ANOVA with post-hoc Tukey adjustment for multiple comparisons.
[3]Standardized values for mRNA expression (mean = 0, standard deviation = 1) centered by control group.
[4]Mean separation insignificant after Tukey adjustment.
[5]Number of samples below threshold: *5HTR3*, 9: *CHRM4*, 5: *CHRNA3*, 1: *CHRNB2*, 1: *DRD1*, 1: *DRD2*, 3: *DRD4*, 18: *GABBR2*, 1: *GLRA4*, 9: *GRIN2C*, 2: *SLC18A2*, 8: *SLC6A2*, 15: *SLC6A3*, 14: *SLC6A4*, 4.
[abc]Means without a common superscript differ (P < 0.05).

Supplemental Table 7. Continued[1]

	Diet								
	CON		OF		OF + 2′FL				
Measure[3]	N	Mean	N	Mean	N	Mean	Pooled SEM	*P*-Value[2]	Accession Number
NR4A2	11	0	11	-0.36	12	-0.41	0.31	0.580	NM_001190276.1
PLP	12	0	12	0.23	11	0.64	0.30	0.314	NM_213974.1
PP1AC	12	0	12	-0.47	12	0.31	0.29	0.141	NM_001044559.1
SIRT1	11	0	11	-0.41	12	0.19	0.30	0.358	NM_001145750.1
SLC17A6	12	0	12	-0.46	12	-0.41	0.31	0.483	XM_003122911.4
SLC17A7	12	0	12	0.13	12	0.36	0.38	0.492	XM_003127311.3
SLC17A8	12	0	12	-0.31	12	0.54	0.28	0.109	XM_003126690.5
SLC18A1	12	0	12	-0.75	11	-0.46	0.29	0.165	XM_013990243.1
SLC18A2[5]	12	0	12	-0.78	12	-0.36	0.29	0.180	NM_001315654.1
SLC18A3	12	0	12	0.59	12	0.72	0.28	0.179	XM_013983446.1
SLC1A1	12	0	12	0.11	12	0.42	0.29	0.579	NM_001164649.1
SLC1A2	12	0	12	-0.18	12	0.24	0.33	0.574	XM_003480708.3
SLC1A3	12	0	12	-0.13	11	0.08	0.30	0.883	NM_001244425.2
SLC1A6	11	0	11	-0.21	12	0.18	0.31	0.613	XM_003123429.4
SLC1A7	12	0	12	-0.52	11	-0.23	0.30	0.440	XM_013999377.1
SLC32A1	11	0	11	-0.41	12	-0.28	0.30	0.599	XM_003134437.4
SLC6A1	12	0	12	-0.17	12	0.02	0.30	0.884	XM_005669778.2
SLC6A11	12	0	12	-0.66	12	-0.10	0.30	0.223	XM_005657080.1
SLC6A13	12	0	12	-0.97	12	-0.51	0.29	0.074	XM_003126576.4
SLC6A2[5]	12	0	12	-0.12	12	-0.72	0.30	0.172	XM_013994585.1
SLC6A3[5]	12	0	12	-0.25	12	-0.28	0.32	0.763	XM_005674638.2
SLC6A4[5]	12	0	12	-0.32	11	-0.06	0.34	0.679	XM_013981192.1
SNAP25	12	0	12	-0.62	12	-0.35	0.30	0.326	XM_003359900.3

[1]Abbreviations: CON, control group; OF, pigs fed oligofructose; OF + 2′FL, pigs fed oligofructose and 2′fucosyllactose; SEM, standard error of the mean.

[2]Data analyzed via two-way ANOVA with post-hoc Tukey adjustment for multiple comparisons.

[3]Standardized values for mRNA expression (mean = 0, standard deviation = 1) centered by control group.

[4]Mean separation insignificant after Tukey adjustment.

[5]Number of samples below threshold: *5HTR3*, 9: *CHRM4*, 5: *CHRNA3*, 1: *CHRNB2*, 1: *DRD1*, 1: *DRD2*, 3: *DRD4*, 18: *GABBR2*, 1: *GLRA4*, 9: *GRIN2C*, 2: *SLC18A2*, 8: *SLC6A2*, 15: *SLC6A3*, 14: *SLC6A4*, 4.

[abc]Means without a common superscript differ (P < 0.05).

Supplemental Table 7. Continued[1]

	Diet								
	CON		OF		OF + 2'FL				
Measure[3]	N	Mean	N	Mean	N	Mean	Pooled SEM	*P*-Value[2]	Accession Number
SYP	12	0	12	-0.01	12	0.26	0.32	0.772	XM_003135078.4
UBE3A	12	0	12	-0.64	12	-0.28	0.29	0.298	NM_001243181.2

[1]Abbreviations: CON, control group; OF, pigs fed oligofructose; OF + 2'FL, pigs fed oligofructose and 2'fucosyllactose; SEM, standard error of the mean.

[2]Data analyzed via two-way ANOVA with post-hoc Tukey adjustment for multiple comparisons.

[3]Standardized values for mRNA expression (mean = 0, standard deviation = 1) centered by control group.

[4]Mean separation insignificant after Tukey adjustment.

[5]Number of samples below threshold: *5HTR3*, 9: *CHRM4*, 5: *CHRNA3*, 1: *CHRNB2*, 1: *DRD1*, 1: *DRD2*, 3: *DRD4*, 18: *GABBR2*, 1: *GLRA4*, 9: *GRIN2C*, 2: *SLC18A2*, 8: *SLC6A2*, 15: *SLC6A3*, 14: *SLC6A4*, 4.

[abc]Means without a common superscript differ ($P < 0.05$).

Supplemental Table 7. Continued[1]

Measure[3]	Diet						Pooled SEM	*P*-Value[2]	Accession Number
	CON		OF		OF + 2′FL				
	N	Mean	N	Mean	N	Mean			
Housekeeping Genes									
ACTB	12	0	12	-0.25	12	0.43	0.31	0.205	XM_003124280.4
GAPDH	12	0	12	-0.16	12	0.22	0.31	0.664	NM_001206359.1
GUS	12	0	12	0.13	12	-0.28	0.29	0.600	NM_001123121.1
HOMER1	11	0	12	-0.68	12	-0.71	0.28	0.149	NM_001243811.1
RPL19	12	0	12	-0.04	12	0.47	0.31	0.335	XM_003131509.4
RPS18	12	0	12	0.58	11	0.64	0.33	0.124	NM_213940.1
TUBB	12	0	12	-0.28	12	-0.41	0.31	0.574	NM_001044612.1

[1]Abbreviations: CON, control group; OF, pigs fed oligofructose; OF + 2′FL, pigs fed oligofructose and 2′fucosyllactose; SEM, standard error of the mean.

[2]Data analyzed via two-way ANOVA with post-hoc Tukey adjustment for multiple comparisons.

[3]Standardized values for mRNA expression (mean = 0, standard deviation = 1) centered by control group.

[4]Mean separation insignificant after Tukey adjustment.

[abc]Means without a common superscript differ (P < 0.05).

Supplemental Table 8. Correlations between the recognition index and significant MRI and gene expression outcomes[1,2]

Measure	CON		OF		OF + 2'FL		Overall	
	r	***P***	***r***	***P***	***r***	***P***	β_1	***P***
1-h Delay								
Olfactory Bulb, %TBV	-0.76	0.03	0.14	0.66	-0.34	0.37	-0.21	0.15
CHRM2	-0.12	0.75	-0.44	0.15	-0.68	0.03	-0.08	0.01
DRD3	-0.34	0.37	-0.28	0.39	0.55	0.10	-0.03	0.45
GABBR1	-0.69	0.04	-0.72	0.01	-0.75	0.02	-0.13	<0.01
HDAC5	-0.78	0.01	-0.29	0.37	-0.65	0.04	-0.11	<0.01
HDAC8	0.10	0.77	-0.37	0.24	-0.04	0.92	-0.06	0.10
NCAM1	0.12	0.76	-0.36	0.24	0.14	0.69	-0.04	0.31
48-h Delay								
Olfactory Bulb, %TBV	-0.76	0.03	-0.65	0.03	0.20	0.61	0.01	0.94
CHRM2	0.32	0.37	0.08	0.82	0.33	0.35	0.01	0.74
DRD3	0.26	0.47	-0.01	0.97	-0.18	0.61	0.04	0.33
GABBR1	-0.36	0.31	0.25	0.46	0.37	0.33	0.04	0.25
HDAC5	-0.55	0.10	0.34	0.31	0.36	0.30	0.07	0.10
HDAC8	0.35	0.32	-0.07	0.84	0.17	0.63	0.02	0.59
NCAM1	0.15	0.68	0.08	0.82	-0.32	0.37	0.01	0.77

[1]Abbreviations: CON, control group; OF, pigs fed oligofructose; OF + 2′FL, pigs fed oligofructose and 2′fucosyllactose; *r;* Pearson correlation coefficient; *P*, P-value; β1, slope of linear regression equation; *DRD3*, dopamine receptor D3; *GABBR1*, GABA type B receptor subunit 1; *HDAC5/8*, histone deacetylases 5 and 8; *NCAM1*, neural cell adhesion molecule 1; *CHRM2*, cholinergic receptor muscarinic 2.

[2]Correlations were performed using only the outcomes significantly affected by diet for MRI and gene expression outcomes against the recognition index after a 1- or 48-h delay. Correlations are presented by diet group, whereas diet was not included in the linear regression model to estimate the diet independent relationship between outcomes. P-values indicate whether the Pearson correlation coefficient or slope are significantly different from zero, indicating a linear relationship between outcomes.
